# Nitric oxide and mitochondria in metabolic syndrome

**DOI:** 10.3389/fphys.2015.00020

**Published:** 2015-02-17

**Authors:** Larisa Litvinova, Dmitriy N. Atochin, Nikolai Fattakhov, Mariia Vasilenko, Pavel Zatolokin, Elena Kirienkova

**Affiliations:** ^1^Laboratory of Immunology and Cellular Biotechnologies, Innovation Park of the Immanuel Kant Baltic Federal UniversityKaliningrad, Russia; ^2^Cardiology Division, Department of Medicine, Cardiovascular Research Center, Harvard Medical School, Massachusetts General HospitalBoston, MA, USA; ^3^Department of Reconstructive and Endoscopic Surgery, Kaliningrad Regional HospitalKaliningrad, Russia

**Keywords:** metabolic syndrome, mitochondrial dysfunction, nitric oxide synthase, nitric oxide, nitric oxide synthase type I, nitric oxide synthase type II, oxide synthase type III, mitochondrial nitric oxide synthase

## Abstract

Metabolic syndrome (MS) is a cluster of metabolic disorders that collectively increase the risk of cardiovascular disease. Nitric oxide (NO) plays a crucial role in the pathogeneses of MS components and is involved in different mitochondrial signaling pathways that control respiration and apoptosis. The present review summarizes the recent information regarding the interrelations of mitochondria and NO in MS. Changes in the activities of different NO synthase isoforms lead to the formation of metabolic disorders and therefore are highlighted here. Reduced endothelial NOS activity and NO bioavailability, as the main factors underlying the endothelial dysfunction that occurs in MS, are discussed in this review in relation to mitochondrial dysfunction. We also focus on potential therapeutic strategies involving NO signaling pathways that can be used to treat patients with metabolic disorders associated with mitochondrial dysfunction. The article may help researchers develop new approaches for the diagnosis, prevention and treatment of MS.

## Introduction

Metabolic syndrome (MS) is a global epidemic, and different environmental, genetic and pathophysiological factors are involved in its development. Mitochondria play central roles in energy metabolism, signaling and apoptosis and its alterations may contribute to the development of metabolic disorders (Peinado et al., 2014). It has been recently found that mitochondrial biogenesis and function are enhanced by nitric oxide (NO), which is a key signaling molecule in vascular homeostasis. This finding indicates that changes in the production of NO bioavailability and MS may be associated with the mitochondria. However, the link between NO signaling components and mitochondria in MS in different tissues is still not clear.

Today, mitochondria remains an attractive target for the prevention and therapy of MS and its complications (Sorriento et al., 2014). Understanding the mechanisms that lead to decreased mitochondrial activity in insulin-sensitive tissues is important for developing therapies to reverse insulin resistance. A deeper understanding of the role of NO pathway components in this process opens up new opportunities for applied research in this area.

## Metabolic syndrome and mitochondrial dysfunction

Mitochondrial dysfunction is closely associated with obesity, metabolic syndrome and type 2 diabetes mellitus (T2DM) (Lowell and Shulman, 2005; Agrawal and Prakash, 2014). Changes in the mitochondrial membrane potential, a reduction in the ATP level, the inhibition of mitochondrial oxygen consumption and reduced mitochondrial biogenesis (Ren et al., 2010) are some of the characteristics of mitochondrial dysfunction (Pieczenik and Neustadt, 2007). The underlying mechanism of mitochondrial dysfunction is very complex, which includes genetic factors from both nuclear and mitochondrial genome and numerous environmental factors (Lee et al., 2010). In addition to these factors, excessive formation of reactive oxygen species (ROS) in obesity and T2DM (Chattopadhyay et al., 2015) contributes to mitochondrial dysfunction, while at physiological levels ROS participate in intracellular signaling and regulation as “redox messengers.” Particularly, superoxide anion generated by the mitochondria, particularly by complexes I and III of the electron transport chain (ETC), is the precursor of most ROS and a mediator in oxidative chain reactions. Dismutation of superoxide produces hydrogen peroxide, which in turn may be partially reduced to hydroxyl radical causing more damage to various mitochondrial and cellular components (Turrens, 2003). Free radical damage to mitochondria may lead to decreased affinity of mitochondrial proteins for substrates or coenzymes (Liu et al., 2002).

The centerpiece of the pathophysiologic mechanism of MS is insulin resistance. The interplay between mitochondrial dysfunction and insulin resistance was first discovered in 1963 (Laguens and Bianchi, 1963). Kitt Falk Petersen et al. demonstrated that insulin resistance, which frequently occurs in elderly individuals, is characterized by a decrease in mitochondrial ATP production, a reduction in mitochondrial RNA, decreased activity of oxidative phosphorylation complexes (Petersen et al., 2003). It is still unclear whether mitochondrial dysfunction results from or causes insulin resistance (Martin and McGee, 2014). One of the key pathophysiological factors underlying the formation of insulin resistance may be the dysregulation of energy metabolism in insulin-sensitive tissues such as skeletal muscle, liver, and adipose (Litvinova et al., 2014). Particularly, in skeletal muscle, decreased mitochondrial respiration capacity, reduced ATP production, and increased ROS levels lead to reduced fatty acid oxidation and increased cytosolic free fatty acid levels, resulting in insulin resistance and T2DM (Pagel-Langenickel et al., 2010). Tg (sm/p22phox) mice, which produce increased vascular ROS, display impaired spontaneous activity and increased mitochondrial ROS production and mitochondrial dysfunction in their skeletal muscle (Youn et al., 2014). As regards liver, there are findings that strongly support that non-alcoholic fatty liver disease can be considered as the hepatic representation of MS (Paschos and Paletas, 2009). Insulin resistance is a key pathogenic factor in both pathologic states. It has been shown that patients with non-alcoholic fatty liver disease had decreased activities of ETC complexes in liver (Pérez-Carreras et al., 2003). Recent findings suggests that reduced SIRT3 activity in fatty liver may result in hyperacetylation of mitochondrial proteins that contributes to mitochondrial dysfunction (Kendrick et al., 2011). Nitrosative stress in non-alcoholic fatty liver disease also contributes to the development of mitochondrial dysfunction that is mediated by the oxidative modifications of mitochondrial DNA (mtDNA), lipids and proteins (Song et al., 2013). Moreover, high-fat diet-induced mtDNA damage correlates with increased oxidative stress in skeletal muscle and liver, which is associated with the induction of markers of endoplasmic reticulum stress, protein degradation and apoptosis (Yuzefovych et al., 2013). In adipose tissue, mitochondria provide key intermediates for the synthesis of triglycerides and are critical for lipogenesis. Adipose mitochondria are also important for lipolysis through the oxidation of fatty acids, which constitutes an important source of ATP to supply cells with energy (Serra et al., 2013). The study of M. Rossmeisl et al. demonstrated that uncoupling of oxidative phosphorylation depresses fatty acid synthesis in white fat (Rossmeisl et al., 2000). The authors show that reduction of adiposity via mitochondrial uncoupling in white fat not only reflects increased energy expenditure, but also decreased *in situ* lipogenesis. Therefore, various studies suggest that modulation of mitochondrial function (including oxidative phosphorylation, ATP synthesis and ROS generation) in above-mentioned tissues may in turn may affect the development of insulin resistance and obesity.

Mitochondrial dysfunction and insulin resistance promote chronic inflammation and the formation of atherosclerotic lesions, and the activation of the inflammatory Toll-like receptor 2/nuclear factor-κB signaling pathway in monocytes is a central mechanism leading to a vicious cycle between chronic inflammation and mitochondrial ROS production (Hulsmans et al., 2012). Tumor necrosis factor (TNF)-α is a key proinflammatory mediator that plays an important role in the chronic inflammation of adipose tissue in obese subjects (Hotamisligil et al., 1993). It has been concluded that TNF-α also induces mitochondrial dysfunction by reducing complex III activity in the ETC, increasing ROS production, and causing damage to mtDNA in cardiac myocytes (Suematsu et al., 2003). It has been demonstrated in mice with adipocytes deficient in mitochondrial transcription factor A that isolated mitochondrial dysfunction in adipose tissue can cause lipodystrophy and lead to MS and chronic inflammation (Vernochet et al., 2014). Thus, mitochondrial alterations that occur during insulin resistance trigger a cascade of pathological processes in different tissues and form a vicious cycle between chronic inflammation and mitochondrial oxidative stress that eventually contributes to cardiovascular pathologies and T2DM.

## Mitochondrial DNA as a molecular marker of metabolic syndrome

MtDNA is a 16569-bp, circular, double-stranded molecule that harbors 37 genes involved in the energy production that takes place in the ETC. This gene set includes 13 structural genes that code for subunits of oxidative phosphorylation complexes as well as genes encoding 22 tRNAs and 2 rRNAs that are involved in the protein synthesis that occurs directly within mitochondria (Smits et al., 2010). According to current estimates, the human genome incorporates approximately 2500 mitochondrial protein-encoding genes; however, only fewer than 1000 of these proteins have been identified in isolated mitochondria (Jiang and Wang, 2012).

It is now established that mtDNA polymorphisms may be risk factors for T2DM and other metabolic pathologies (Nishigaki et al., 2011). The substitution 16189T>C in the D-loop region of mtDNA has been shown to be associated with insulin resistance, obesity and T2DM in both European (Poulton et al., 2002) and Asian (Kim et al., 2002) populations. Analysis of mtDNA has also shown that haplogroup N9A may be a protective factor against MS in Japanese women (Tanaka et al., 2007) and against T2DM in Japanese and Korean individuals (Fuku et al., 2007). Furthermore, mutations in mtDNA have been independently associated with multiple phenotypes of MS. The T4291C mutation in mtDNA has been shown to be associated with MS in a large Caucasian family, and a common mtDNA variant, T16189C, has been shown to be associated with a lower body mass index at birth, insulin resistance, dilated cardiomyopathy and an increased susceptibility to MS in a Chinese population (Palmieri et al., 2011). Moreover, the mtDNA mutation A8890G has been found in a patient with juvenile-onset MS (Ye et al., 2013).

Changes in the amount of mtDNA may be a consequence of genetic defects (Rötig and Poulton, 2009) and hormonal imbalances in non-hereditary diseases (Liu et al., 2003). Our results showing a reduced number of mtDNA copies in peripheral blood and samples of adipose tissue obtained from different locations from patients with MS (Mozhey et al., 2014) are consistent with the results of previous studies. It has been previously shown that decreased mtDNA content in peripheral blood occurs before the onset of T2DM (Lee et al., 1998). The administration of pioglitazone, which is a mainstay drug for the treatment of T2DM, leads to an increase in the number of mtDNA copies in subcutaneous adipocytes *in vitro* (Bogacka et al., 2005). As previously mentioned, mtDNA levels may vary in different organs. However, experiments performed in rats have shown that changes in mtDNA copy number in peripheral blood leukocytes reflect similar processes in muscle tissue and hepatocytes. Thus, we can assume that the level of mtDNA in human peripheral blood cells is an indicator of various metabolic disorders. Moreover, the quantitative estimation of mtDNA in various biological samples might become a tool for the prognosis and estimation of treatment efficiency of MS.

## Interconnections between mitochondria and nitric oxide production in different tissues

NO is a relatively stable gas that belongs to a family of gas transmitters with similar inherent intracellular effects. NO is a regulator of many physiological processes. It is synthesized by vascular endothelial cells, is responsible for vasodilatation and is involved in various processes in the nervous, reproductive and immune systems. NO prevents platelet aggregation and the adhesion of neutrophils to the endothelium, and it has cytostatic and cytotoxic properties (Ufnal and Żera, 2010). In addition to its participation in the regulation of vascular smooth muscle tone, NO directly affects mitochondrial respiration (Lee, 2000). Its inhibition of ETC enzymes may also be one of the causes of the decrease in oxygen consumption by cardiomyocytes. NO plays important roles in the development of MS components, such as insulin resistance, endothelial dysfunction, hypertriglyceridemia and chronic adipose tissue inflammation.

NO is formed from L-arginine, when in the presence of oxygen, by isoenzymes of nitric oxide synthases (NOS), which depend on the cell type. Endothelial NOS (eNOS) and neuronal NOS (nNOS) are constantly active and produce small amounts of NO in response to stimuli that induce an increase in the intracellular Ca^2+^ concentration (Förstermann and Sessa, 2012). Inducible NOS (iNOS), which is a calcium-independent form of NOS, is localized to macrophages, neutrophils, and micro- and astroglial cells and is expressed under the influence of factors such as cytokines and bacterial lipopolysaccharide (Yang et al., 2014). The mitochondrial production of NO is catalyzed by mitochondrial NOS (mtNOS), which has been identified as the alpha isoform of nNOS that is acylated at a Thr or Ser residue and phosphorylated at the C-terminus.

Impaired NOS activity is closely associated with insulin resistance (Kashyap et al., 2005). The work of Nisoli's group has firmly established the function of NO generated by eNOS and nNOS as an upstream regulator of mitochondrial biogenesis (Nisoli et al., 2003). Experiments with homozygous eNOS knockout mice have definitively proven the relationship between NO and insulin sensitivity because these mice show increased blood pressure and insulin resistance (Shankar et al., 2000).

**Figure 1:**
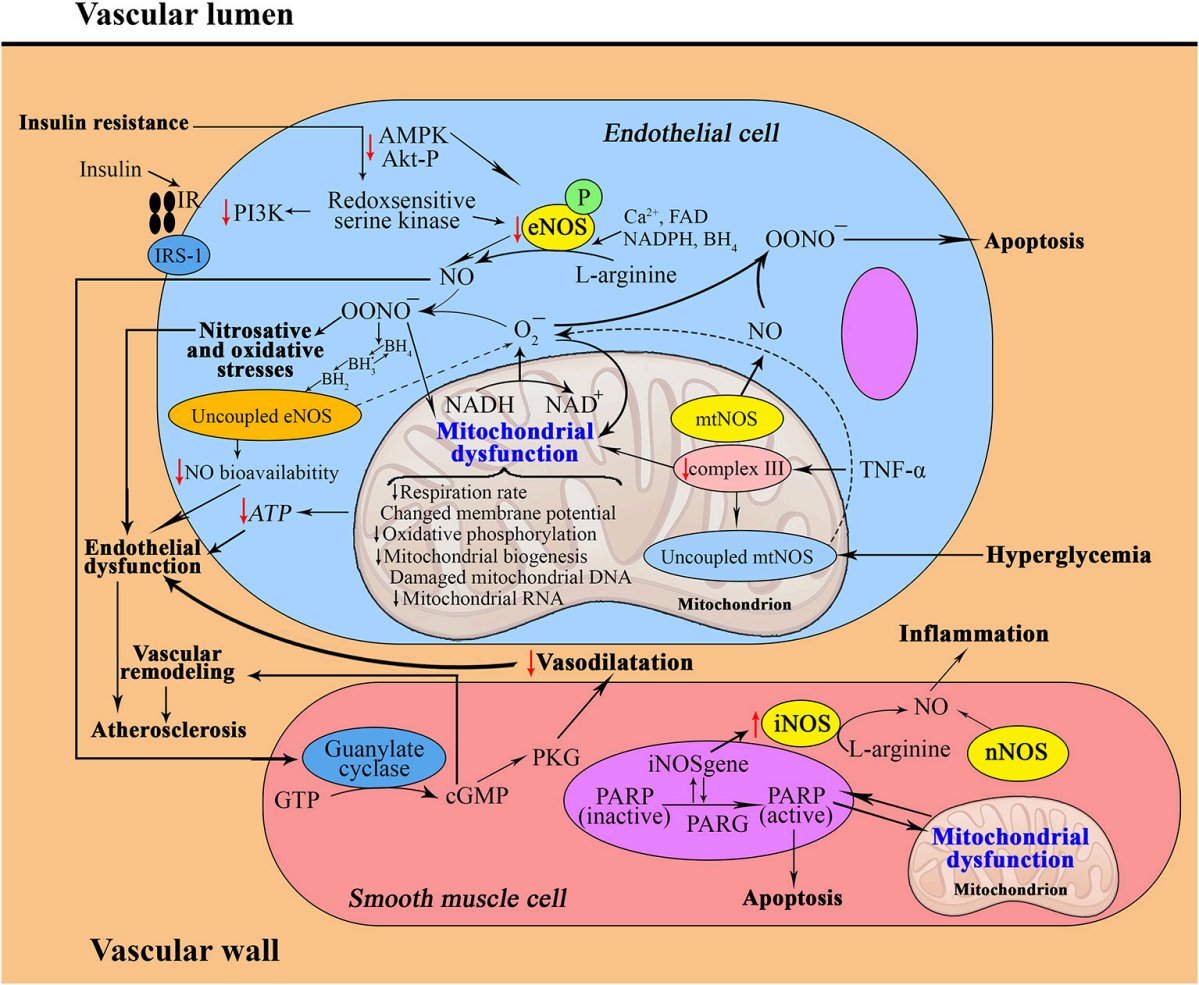
**Interrelation between nitric oxide and mitochondria in the pathophysiology of metabolic syndrome (a, in the vascular wall; b, in an adipocyte; c, in a hepatocyte)**. a: Insulin resistance contributes to decreased activities of signaling molecules, which leads to decreased eNOS activity. Superoxide generated in mitochondria can modestly, and peroxynitrite can strongly, oxidize BH4–BH2, leading to uncoupling of eNOS, increased production of ROS and endothelial dysfunction. Moreover, mtNOS becomes “uncoupled”; it switches from a NO-generating enzyme to a superoxide-producing enzyme. Increased production of peroxynitrite and superoxide anions generated from uncoupled mtNOS and eNOS damage mitochondrial ETC complexes, leading to mitochondrial dysfunction. eNOS then generates superoxide rather than NO, which contributes to vascular oxidative stress and further reduces NO bioavailability. Excessive mtNOS-derived NO can produce reactive nitrogen species, such as peroxynitrite, which may cause tyrosine nitration of mitochondrial components and may play a key role in apoptosis. Under the conditions of oxidative stress, the enhanced activity of iNOS, with less contribution of nNOS, in vascular smooth muscle cells induces the development of chronic metabolic inflammation.

Endothelial dysfunction, which is defined by a decrease in flow-mediated vasodilatation and vascular insulin resistance, represents an established vascular abnormality in diabetic patients (Montagnani et al., 2001). The possible underlying mechanisms include reductions in the levels of eNOS mRNA expression and protein production (Huang, 2009). In particular, Alvarado-Vásquez et al. have shown that human umbilical vein endothelial cells isolated from healthy newborns with a strong family history of T2DM exhibit abnormal intracellular synthesis of NO and impaired expression of the eNOS, GLUT1 and p53 genes, which are all associated with NO synthesis (Alvarado-Vásquez et al., 2007). However, studies in animal models and in humans have assumed that T2DM and atherosclerosis are not necessarily associated with reductions in total eNOS (Felaco et al., 2001). Several mechanisms leading to endothelial dysfunction, such as a lack of enzymatic cofactors for eNOS (Mangge et al., 2014), the inactivation of NO by the reaction of superoxide (O2-) resulting in peroxynitrite anion (OONO-) formation (Pacher et al., 2007), the influence of superoxide NADPH oxidase (Griendling et al., 2000) and “uncoupled” eNOS, have been demonstrated (Figure 1). Peroxynitrite, in turn, has been shown to uncouple endothelial nitric oxide synthase (eNOS), thereby converting an antiatherosclerotic NO-producing enzyme into an enzyme that may accelerate the atherosclerotic process by producing superoxide (Schulz et al., 2008). It should be noted that these mechanisms are not mutually exclusive and can occur simultaneously. The studies of Youn et al. have demonstrated that the p47phox and NOXO1-dependent activation of NOX1, but not of NOX2, NOX4 or mitochondria, mediates the diabetic uncoupling of eNOS (Youn et al., 2012). Interestingly, NOX1-null mice are protected from diabetic endothelial dysfunction, and mice lacking components of NOX2 are protected against hypertension and show reduced atherosclerotic lesion formation when crossed with the apoE-/- background (Judkins et al., 2010).

ROS derived from mitochondria induce the infiltration and activation of inflammatory cells and increase the apoptosis of endothelial and vascular smooth muscle cells (Hulsmans et al., 2012). Excessive formation of peroxynitrite, which is a reactive nitrogen species, has been demonstrated to accelerate the atherosclerotic process by causing direct structural damage and further ROS production (Münzel et al., 2010). Peroxynitrite also damages DNA and thus triggers the activation of DNA repair systems. A DNA nick-sensor enzyme, poly(ADP-ribose) polymerase-1 (PARP-1), also becomes activated upon sensing DNA breakages. Activated PARP-1 cleaves NAD(+) into nicotinamide and ADP-ribose and polymerizes the latter onto nuclear acceptor proteins. The peroxynitrite-induced overactivation of PARP leads to the consumption of NAD(+) and consequently, ATP, culminating in cell dysfunction, apoptosis or necrosis (Virág et al., 2003). This mechanism of cell death has been implicated in the molecular aspects of T2DM-associated cardiovascular disease.

Recent data have indicated the possibility of a significant role for interrelations between PARP-1 and iNOS in human atherosclerotic lesions. The induction of PARP-1 within the nuclei of lesional smooth muscle cells has been correlated with alterations in mitochondrial morphology (Perrotta et al., 2011). Therefore, it should be concluded that plaque formation is one of the consequences of the PARP-1-mediated mechanism that is involved in changes in mitochondrial morphology.

It has been reported that deletion of eNOS or the pharmacological inhibition of NOS impacts baseline and exercise-stimulated vascular mitochondrial biogenesis and dynamics (Miller et al., 2013). In particular, in cultured human aortic endothelial cells, a similar loss of mitochondrial networks and increased expression of mitochondrial fission 1 protein (Fis1) and dynamin-related protein-1 (Drp1), which are proteins required for mitochondrial fission, have been demonstrated. The silencing of Fis1 or Drp1 expression by siRNA reduces high glucose-induced alterations in mitochondrial networks, ROS production, eNOS activation, and cGMP production (Shenouda et al., 2011). The increased expression of Fis1 and Drp1 and decreased expression of fusion proteins (Opa1, Mfn1, and Mfn2) in addition to decreases in cAMP response element binding protein and PGC-1α have also been observed in aortic tissues of eNOS-null rodents (Miller et al., 2013). These data suggest that NOS is a biologically important modulator of basal vascular mitochondrial function and structure.

**Figure 2:**
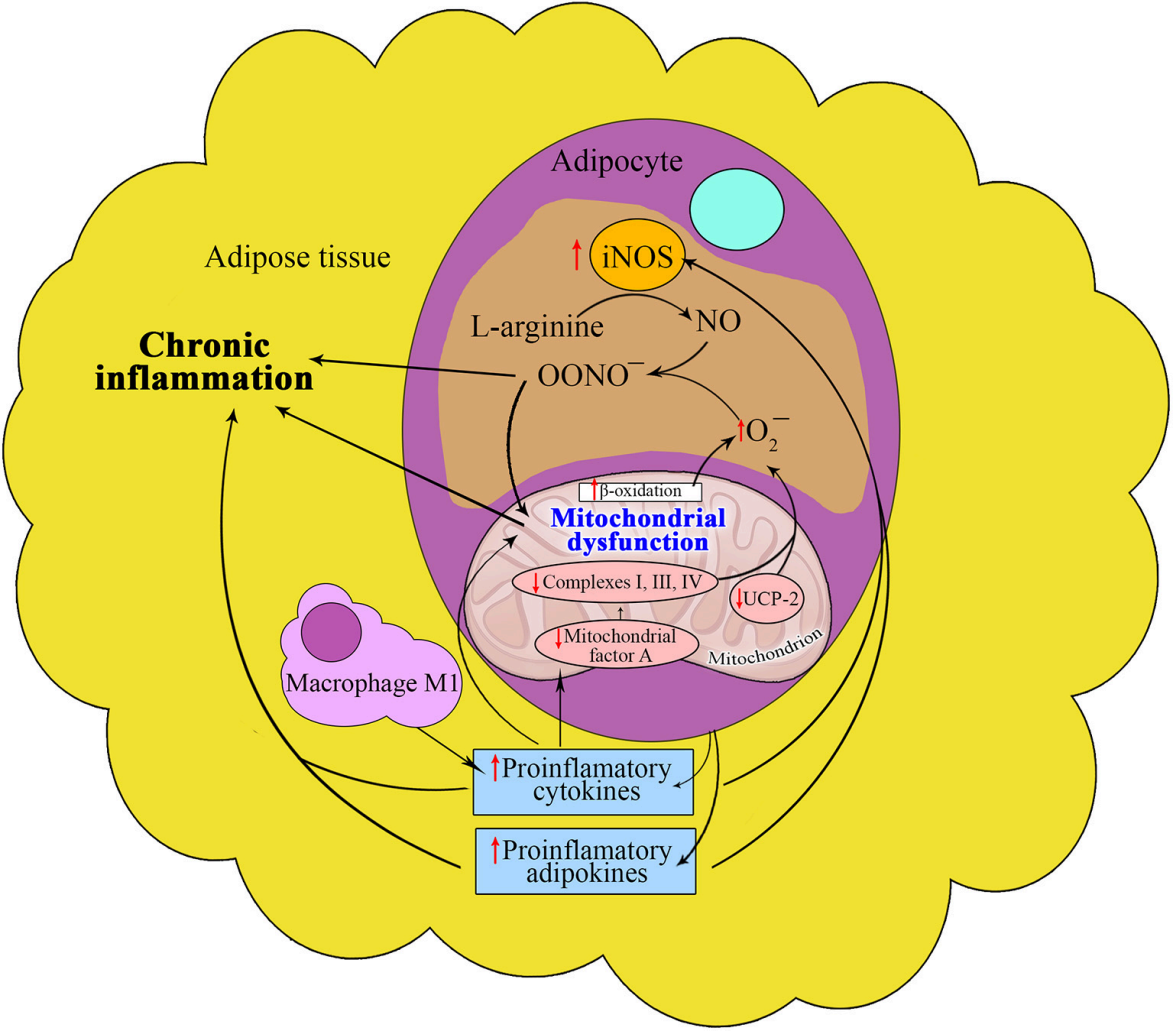
**Interrelation between nitric oxide and mitochondria in adipose tissue in metabolic syndrome**. The proinflammatory microenvironment of adipose tissue induces the iNOS activity in MS that increases peroxynitrite synthesis, thus contributing to oxidative stress and chronic inflammation. Under the influence of these conditions the reduced expression of the mitochondrial protein UCP-2 promotes the formation of MS components through decreased NO bioavailability and increased ROS generation. Reduction in the expression of mitochondrial transcription factor A also contributes to the development of MS components.

Enhanced iNOS gene expression and production in different tissues, especially in insulin-sensitive tissues, leads to peroxynitrite formation, causing nitrosative and oxidative stresses. Recent studies have shown the involvement of TNF-α-induced iNOS in the expression of mitochondrial protein uncoupling protein-2 (UCP-2) in adipocytes (Merial et al., 2000) as well as in NO bioavailability in adipose tissue (Tian et al., 2012) (Figure 2). It should be concluded that plaque formation is one of the consequences of the PARP-1-mediated mechanism involved in changes in mitochondrial morphology.

**Figure 3:**
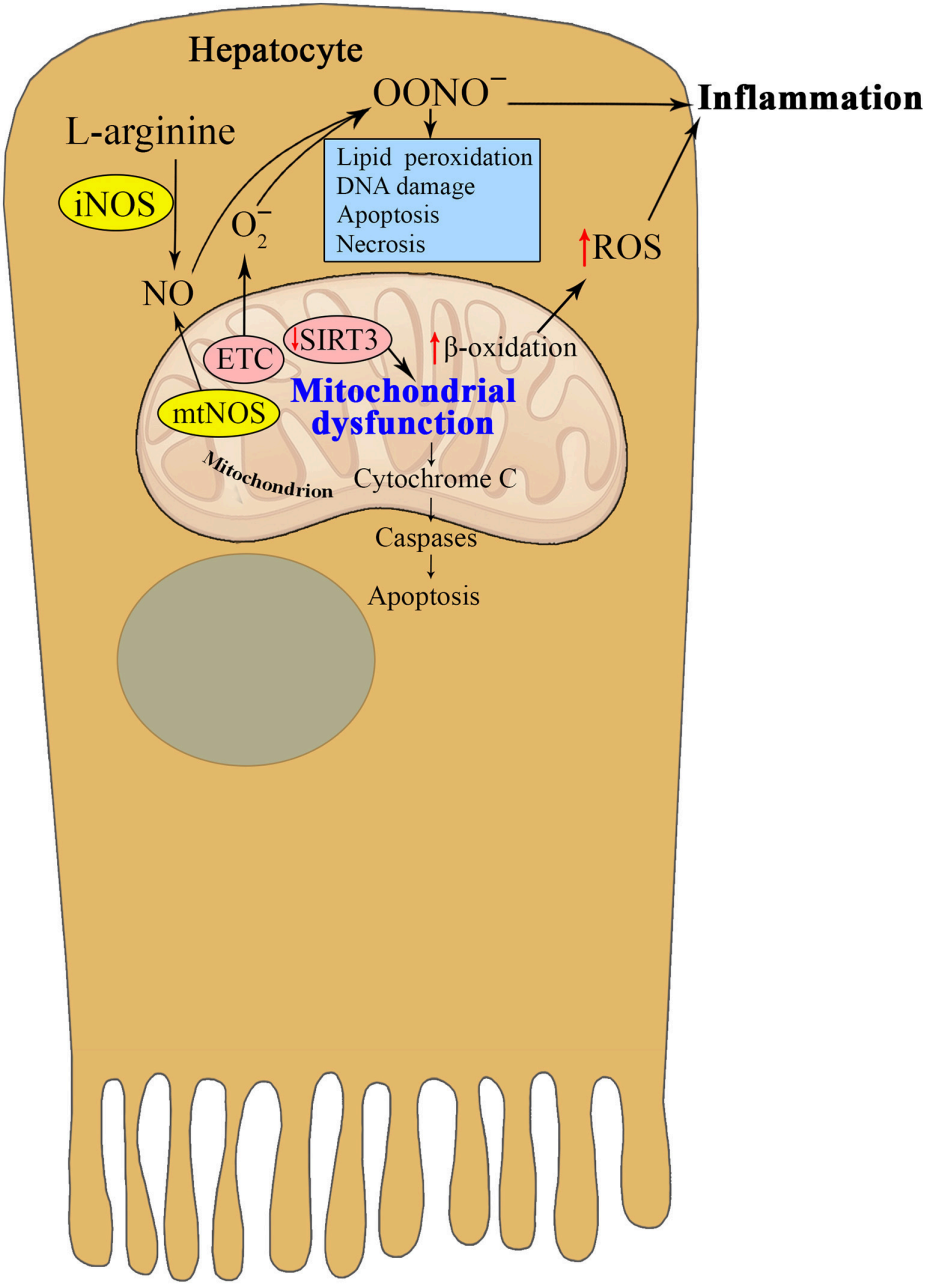
**Interrelation between nitric oxide and mitochondria in hepatocytes in metabolic syndrome**. The enhanced activities of mtNOS and iNOS in hepatocytes during insulin resistance promote peroxynitrite production and cytochrome c release, which are associated with the peroxidation of mitochondrial lipids and apoptosis. Reduced SIRT3 activity contributes to mitochondrial dysfunction, which in turn promotes oxidative stress and chronic inflammation in MS. Taken together, the reviewed studies suggest that NO and the different NOS isoforms play an important role in critical pathological states such as endothelial dysfunction and peroxynitrite-induced cytotoxicity.

Since it was first reported in 1995, the existence of mtNOS has been controversial (Giulivi et al., 1998). It has also been reported that the addition of calcium to mitochondria isolated from hepatocytes stimulates mtNOS, causing peroxynitrite production and cytochrome c release, which are associated with the peroxidation of mitochondrial lipids (Ghafourifar et al., 1999) (Figure 3). Mitochondrial lipid peroxidation may be an important event in mitochondria-mediated apoptosis (Nomura et al., 1999), but there is no direct evidence that lipid peroxidation mediates NO-induced apoptosis. The NO-synthesizing capacity of mtNOS is higher than all of the cytoplasmic NOS isoforms combined (Brookes, 2004). The discovery of Ca-dependent mtNOS was an outstanding achievement that elucidated late aging-related metabolic alterations that had taken years to become apparent (Ghafourifar and Cadenas, 2005). The participation of mtNOS appears to be of primary importance but remains to be fully elucidated.

## Potential therapeutic targets affecting mitochondrial dysfunction and the nitric oxide pathway in metabolic syndrome

Different research groups have studied various ways to regulate NO signaling pathways to modulate mitochondrial dysfunction. NO signaling in mitochondria underlies many of the metabolic effects of NO, and at low physiologic levels, links the cellular energy demand with the mitochondrial energy supply, while beneficially affecting mitochondrial oxidative stress and calcium handling (Levine et al., 2012).

Increasing the mitochondrial concentrations of antioxidant drugs by the selective targeting of mitochondria should represent a practical approach for the treatment of a wide range of human diseases. The stimulation of cultured cardiac myocytes with TNF-α or angiotensin II increases ROS generation and myocyte hypertrophy, and treatment with antioxidants inhibits both of these effects (Nakamura et al., 1998). Mitochondria-targeted antioxidants have been developed as pharmaceuticals and have been shown to be safe and effective in phase II clinical trials. Despite these facts and a number of pre-clinical and clinical lines of evidence, the results of studies assessing the effects of classical antioxidants, such as vitamin C, vitamin E, or folic acid in combination with vitamin E, have been disappointing (Münzel et al., 2010).

Medications used to treat T2DM focus on reducing blood glucose levels. Oral antihyperglycemic medications are used in T2DM to increase organ sensitivity, increase insulin secretion, or decrease glucose absorption during digestion. Insulin and metformin represent the first-line treatments for T2DM (Blake and Trounce, 2014), and their effects on mitochondrial function may contribute to the mechanism underlying dysfunction in T2DM. Different hypoglycemic agents, and exercise may reduce oxidative stress in diabetic patients through reducing NOX activity or improving mitochondrial function, which may prevent or postpone the development of cardiovascular complications (Shen, 2010). Moreover, substances such as statins, angiotensin-converting enzyme inhibitors, or AT1-receptor blockers, which possess indirect antioxidant properties mediated by the stimulation of NO production and by the simultaneous inhibition of superoxide production, have been shown to improve vascular function in pre-clinical and clinical studies and to reduce the incidence of cardiovascular events in patients with cardiovascular disease (Münzel et al., 2010). These drugs and the NO-releasing β-blocker nebivolol have been shown to possess combined antioxidant properties (Münzel and Gori, 2009). Evidence is emerging in support of improved NO bioavailability as a result of exercise training due to its enhanced synthesis and to a reduction in its oxidative stress-mediated destruction. Molecular targets sensitive to the effects of exercise training include eNOS and the antioxidant enzyme superoxide dismutase. However, many fundamental details of the cellular and molecular mechanisms linking exercise to alterations in molecular and functional endothelial phenotypes remain to be discovered (Rush et al., 2005). Thus, the ideal approach to antioxidant therapy requires the stimulation of NO production and the simultaneous inhibition of vascular superoxide production.

Targeting NOS uncoupling to reduce oxidative stress is also possible in humans through the supplementation of the NOS cofactor tetrahydrobiopterin (BH4) or of its precursor sepiapterin. BH4 has been shown to improve endothelial dysfunction in smokers, diabetic subjects (Heitzer et al., 2000) and in patients with hypertension, hypercholesterolemia or coronary artery disease (Schulz et al., 2008). Folate, which has mild antioxidant properties, can reverse NOS uncoupling, possibly via BH4-dependent mechanisms (Verhaar et al., 2002). Despite these pre-clinical data, the effects of BH4 and related molecules on cardiovascular morbidity and mortality remain unknown, although the possibility of an improved cardiovascular prognosis due to folate supplementation has been definitively ruled out by several randomized controlled trials (Bazzano et al., 2006).

Instead of preventing NO degradation through reducing oxidative stress, it may be possible to provide exogenous NO via administering NO-donating substances, such as organic nitrates. However, research has shown that these mediators cause endothelial dysfunction and increase oxidative stress through at least three different mechanisms, including uncoupling of the ETC, activation of ROS-producing enzymes (including NOS uncoupling), and the direct reaction of nitrate-derived NO with vascular superoxide to form ONOO_2_ (Münzel et al., 2005). In turn, these mechanisms accelerate, rather than inhibit, oxidative stress.

Recent evidence has suggested that coenzyme Q10 supplementation may be useful to treat obesity, oxidative stress and the inflammatory process in MS patients because Co–Q10 is an essential component of the ETC. The anti-inflammatory response and lipid-metabolizing effects of coenzyme Q10 are likely mediated by transcriptional regulation of inflammation and lipid metabolism (Alam and Rahman, 2014). Curcumin also represents a potential antioxidant and anti-inflammatory agent. Studies have demonstrated that curcumin treatment increases oxygen consumption and significantly decreases lipid and protein oxidation levels in liver mitochondria isolated from high-fat diet-induced obese mice compared with untreated obese mice (Martínez-Morúa et al., 2013). Furthermore, curcumin treatment does not cause body weight gain or affect mitochondrial NO synthesis. These findings suggest that obesity induces oxidative stress and mitochondrial dysfunction, whereas curcumin may confer protection against these effects.

Ecdysterone treatment normalizes the levels of stable NO metabolites in addition to the activities of superoxide, iNOS and nNOS in mitochondria in the heart of rats with streptozotocin-induced diabetes (Korkach et al., 2007). These results suggest that ecdysterone treatment attenuates diabetes-induced mitochondrial alterations and protects against oxidative and nitrosative stresses. A study of insulin-resistant male Zucker obese rats by Aroor et al. has shown that NADPH oxidase inhibitors (apocynin and VAS2870) and BH4 fail to modify bradykinin-induced vasodilatation. This group has shown that superoxide generation is elevated in vessels of insulin-resistant, morbidly obese subjects and reduced by the mitochondrial-targeted superoxide dismutase mimetic mito-TEMPO (El Assar et al., 2013). Vasodilatation has been shown to improve in insulin-resistant morbidly obese arteries as a result of the activity of the superoxide scavengers, superoxide dismutase and mito-TEMPO (Aroor et al., 2013). The blockade of TNF-α with infliximab, but not the inhibition of iNOS or cyclooxygenase, improves endothelial relaxation and decreases superoxide formation.

Endoplasmic reticulum stress has been implicated in the mitochondrial dysfunction-induced apoptosis of pancreatic beta cells. Furthermore, mitochondrial dysfunction increases the expression of the iNOS gene and the production of NO, but NO production is inhibited by compound C or by the dominant-negative mutant form of AMP-activated protein kinase (AMPK) K45R (Shen, 2010). Moreover, treatment with 1400W, an inhibitor of iNOS, prevents endoplasmic reticulum stress and apoptosis induced by mitochondrial dysfunction (Shen, 2010). Interestingly, the NO donor S-nitroso N-acetylpenicillamine, the nonspecific NOS inhibitor NG-nitro-L-arginine methyl ester, and the specific nNOS inhibitor 1-(2-trifluoromethylphenyl) imidazole significantly reduce creatine kinase leakage, cell necrosis, and apoptosis in diabetic myocardium (Barua et al., 2011). These results demonstrate that both the provision of exogenous NO and the suppression of endogenous NO production result in the potent protection of diabetic human myocardium, overcoming the unresponsiveness of these tissues to cardioprotective therapies. The treatment of cells with the NG-nitro-L-arginine methyl ester (1 mmol/l) has been shown to significantly diminish the TNF-α-mediated sustained downregulation of UCP-2 expression, whereas treatment with the NO donor S-nitroso-L-glutathione (1 mmol/l) mimics the effect of TNF-α on UCP-2 expression (Merial et al., 2000).

The modulation of NO-mediated pathways through dietary supplementation with L-arginine or its precursor L-citrulline may represent an effective and practical strategy to prevent and treat MS, including obesity, T2DM, and dyslipidemia, in mammals such as humans (Jobgen et al., 2006). Clinical research evaluating the effects of arginine and citrulline in mitochondrial diseases has been limited to uncontrolled, open-label studies, which have demonstrated that administering arginine to subjects with mitochondrial encephalomyopathy, lactic acidosis or stroke-like episodes (MELAS) syndrome results in improvement of the clinical symptoms associated with the stroke-like episodes and decreased frequency and severity of these episodes (El-Hattab et al., 2012).

Pathways that regulate mitochondrial biogenesis have recently emerged as potential therapeutic targets for the amelioration of the endothelial and vascular dysfunction observed in metabolic diseases (Schulz et al., 2008). Shen et al. have suggested that lipoamide is a potent stimulator of mitochondrial biogenesis in adipocytes and may have potential therapeutic applications in obesity and T2DM (Shen et al., 2010). Lipoamide dose-dependently stimulates the expression of eNOS and the formation of cGMP. In addition, knockdown of eNOS using siRNA prevents lipoamide-induced mitochondrial biogenesis, which is also blocked by the soluble guanylate cyclase inhibitor 1H-[1,2,4]oxadiazolo[4,3-a]quinoxalin-1-one and by the protein kinase G inhibitor KT5823. Thus, the stimulation of mitochondrial biogenesis by lipoamide involves signaling via the eNOS-cGMP-PKG pathway.

Enhanced NO production is known to activate SIRT1, which is a histone deacetylase that regulates PGC-1α, a regulator of mitochondrial biogenesis and a coactivator of transcription factors that impacts energy homeostasis. Tadalafil-treated diabetic mice exhibit significantly improved left ventricular function that is associated with increased cardiac SIRT1 activity (Koka et al., 2014). Tadalafil also enhances plasma NO oxidation levels, myocardial SIRT1, PGC-1α expression, and the phosphorylation of eNOS, Akt, and AMPK in diabetic hearts. Oxidative phosphorylation of the complex I substrate glutamate is decreased by 50% in diabetic hearts compared with non-diabetic controls. Tadalafil protects oxidative phosphorylation and improves glutamate state 3 respiration rates. Additionally, the increase in ROS production from complex I is significantly decreased by treatment with tadalafil (Koka et al., 2014).

Studies of resveratrol treatment in primary human coronary arterial endothelial cells have shown that resveratrol induces mitochondrial biogenesis in these cells. The formation of new mitochondria is associated with activation of SIRT1, upregulation of eNOS, and induction of specific mitochondrial biogenesis factors (Schulz et al., 2008). It has also been shown that resveratrol treatment induces mitochondrial biogenesis in the aorta in T2DM mice (Schulz et al., 2008).

The insulin-sensitizing drug pioglitazone is a ligand of PPARγ that ameliorates the basic problem of insulin resistance and is therefore considered to reduce the risk of cardiovascular disease in patients with T2DM (Yki-Järvinen, 2004). Pioglitazone reduces myocardial infarct size via the activation of the PPARγ, phosphoinositide 3-kinase, Akt, and eNOS pathways but not via opening of the mitochondrial K(ATP) channels. The use of this drug may be a novel strategy for the treatment of T2DM with coronary artery disease.

Thus, a deeper understanding of the role of NO and NOS isoforms in mitochondrial pathology is necessary for the study of MS pathogenesis as well as its complications and clinical applications.

## Conclusions

The main components of MS, particularly visceral obesity, insulin resistance, and T2DM, are associated with changes in mitochondrial metabolism in different tissues. Mitochondrial dysfunction may accelerate the progression of insulin resistance and subsequent organ dysfunction and has different interconnections with eNOS, iNOS, nNOS and mtNOS. The main mechanisms involved in MS development include augmented generation of mitochondrial superoxide that is formed with the participation of NO and disruptions in mitochondrial respiration and biogenesis that lead to apoptosis and other consequences. In addition, alterations in mitochondrial activity that occur in MS may include acquired or inherited reductions in the mitochondrial oxidative phosphorylation capacity, submaximal ADP-stimulated oxidative phosphorylation and plasticity of mitochondria and/or reductions in mitochondrial contents in insulin-sensitive tissues.

Currently, mitochondria remain an attractive target for the prevention and treatment of metabolic disorders. Therapy based on the inhibition of iNOS, on the prevention of eNOS uncoupling and on other effects on the components of the NO pathway may help to protect against mitochondrial dysfunction associated with metabolic disorders. Despite great advances in this area, questions remain about the relationships between mitochondrial function and the NOSs in various tissues, such as skeletal muscle, liver, heart and adipose tissues, particularly with regard to the effects of therapeutic strategies that affect mitochondrial function and insulin sensitivity.

### Conflict of interest statement

The authors declare that the research was conducted in the absence of any commercial or financial relationships that could be construed as a potential conflict of interest.
